# Transition from childhood to adulthood in Duchenne muscular dystrophy (DMD)

**DOI:** 10.1186/1750-1172-7-S2-A8

**Published:** 2012-11-22

**Authors:** Sunil Rodger, Birgit F Steffensen, Hanns Lochmüller

**Affiliations:** 1 TREAT-NMD, Institute of Genetic Medicine, Newcastle University, International Centre for Life, Central Parkway, Newcastle upon Tyne, NE1 3BZ, UK; 2 RehabiliteringsCenter for Muskelsvind, Neuromuscular Department, Kongsvang Allé 23, DK-8200 Århus, Denmark

## 

Duchenne muscular dystrophy (DMD) is the most common childhood muscular dystrophy, affecting 1 in 3500 live male births. Mutations in the X chromosome result in an absence of dystrophin, causing progressive muscle degeneration and loss of ambulation by the early teens with respiratory, orthopaedic and cardiac complications. Without intervention these complications lead to death at a mean age of 19 years. However, the natural history of DMD is well-known and can be changed with proactive multidisciplinary management to address predictable complications[1]. Better care has led to a growing adult DMD population, challenging the notion of DMD as a “paediatric” disease. This population faces particular challenges, not only medical (e.g. associated with long-term steroid usage, orthopaedic, ventilation, and cardiac, gastrointestinal or genitourinary problems), but those associated with wider issues of transition. These include medical transfer from paediatric to adult services, and social transition to independent living and full societal inclusion.

Transfer arrangements to adult facilities, which vary considerably between clinics and countries, are usually needed due to regulations governing access to paediatric services. As DMD requires co-ordinated care, this move from cohesive paediatric clinics to disjointed adult services is often problematic, and a successful transfer should be the culmination of a period of planned transition. Wider social transition, enabling independent living and further education/employment, is also very important[2]. However, as with many other disabilities, adults with DMD face obstacles to full participation. Planning is crucial, and preparation for adulthood should be considered in partnership with families as part of a comprehensive package of psychosocial care from diagnosis.

Recent research suggests that despite legal and health frameworks, DMD transition care in the UK is highly diverse and sometimes lacking[3]. Positive experiences were characterised by forward planning and long-standing relationships between the family and healthcare professionals. In Denmark an integrated model of care is provided by the National Rehabilitation Centre for NMDs (RCfM), which supports families from diagnosis with a comprehensive programme of courses and interventions at significant life milestones[4]. Patient advocacy groups also play a very important role in transition, particularly through programmes such as the MDA Transitions Center.

Although there is no one-size-fits-all model for DMD transition care, some features seem particularly important to successful transitions. These include continuity and stability in care; the integration of wider social issues; the involvement of the young man and his family in decision-making; and the support of patient advocacy groups.
